# The nature and rate of cognitive maturation from late childhood to adulthood

**DOI:** 10.3389/fpsyg.2015.00704

**Published:** 2015-05-27

**Authors:** Jason A. Cromer, Adrian J. Schembri, Brian T. Harel, Paul Maruff

**Affiliations:** ^1^Child Study Center, Yale UniversityNew Haven, CT, USA; ^2^Cogstate, Inc.New Haven, CT, USA; ^3^Psychology, Royal Melbourne Institute of Technology UniversityMelbourne, VIC, Australia; ^4^Florey Institute for Neuroscience and Mental Health, University of MelbourneMelbourne, VIC, Australia

**Keywords:** cognition, cognitive development, adolescent development, repeat assessment, neuropsychology, neuropsychological test

## Abstract

To better understand the nature and rate of cognitive change across adolescence, the Cogstate Brief Battery (CBB) was utilized to assess psychomotor function, attention, working memory, and visual learning in individuals aged 10–18 years old. Since all CBB tasks have equivalent perceptual, motor, and linguistic demands as well as being appropriate for both children and adults, this approach allowed direct across-age comparison of multiple cognitive domains. Exponential decreases in reaction time and linear increases in accuracy were observed across adolescent development in a cross-sectional sample of 38,778 individuals and confirmed in a 5788 individual longitudinal sample with 1-year repeat assessments. These results have important implications for the repeated assessment of cognition during development where expected maturational changes in cognition must be accounted for during cognitive testing.

## Introduction

Cognition changes continuously from childhood through to adulthood reflecting maturation of the central nervous system (Gogtay et al., 2004; Casey et al., 2005; Scherf et al., 2006; Brenhouse and Andersen, 2011). By 10 years of age, children have undergone substantial development in cognition likely corresponding to the brain reaching near adult size and weight by that time (Caviness et al., 1996; Luciana and Nelson, 2002; Waber et al., 2007; Luna, 2009). However, even without further increase in brain volume, cognitive functions continue to develop throughout adolescence into adulthood most likely reflecting a refinement of the neural networks that support the more specialized aspects of cognition that characterize adulthood (Bunge and Wright, 2007). The rate of development in cognitive functions also varies across different cognitive domains. For example, simple reaction time may reach adult levels in early adolescence. Conversely, executive functions, which include the ability to integrate multiple cognitive processes in pursuit of a behaviorally relevant goal, continue to mature into adulthood (Paus, 2005; Conklin et al., 2007; Thomas et al., 2011). Because of these characteristics, neuropsychological models of brain disruption in late childhood, adolescence, and early adulthood must be based on a sound understanding of normal cognitive development (Andersen, 2003; Steinberg, 2005; Paus et al., 2008).

One import component to understanding typical cognitive development is measuring the rate of cognitive change that occurs over time with maturation. However, it is currently not clear whether age-related improvement in cognition occurs at a constant rate (i.e., linearly) from childhood to adulthood or whether it slows as age increases (Hale, 1990; Kail, 1993; Luna et al., 2004; Brenhouse and Andersen, 2011). Because the cognitive tasks used to model changes in different cognitive functions across development are often themselves different, it is possible that the varying developmental trajectories that have been observed reflect maturational differences in perceptual, motor or linguistic abilities of children in addition to, or instead of, the cognitive construct under investigation (Thomas et al., 2012). Therefore, additional studies to examine cognitive change across development using cognitive tasks that can be applied across the entire study age range and directly compared against one another would be useful to help characterize normal cognitive maturation.

A second important question regarding typical cognitive development is whether or not the rate of cognitive change over time varies between males and females. Neural development occurs at different times for males and females (Brenhouse and Andersen, 2011), so cognitive development may differ as well. Gender differences in cognitive development have typically been shown for verbal and spatial abilities; however, they have also been reported for other cognitive domains, such as processing speed (Ardila et al., 2011). While the existence of gender differences on cognitive development is controversial, additional studies with large sample sizes would be helpful to address this issue (Ardila et al., 2011).

One reason that different findings may exist regarding rates of cognitive maturation and gender effects during cognitive development is that most studies on these issues have used cross-sectional designs and relatively small sample sizes (e.g., age cohorts of *n* = 10–100). In studies of cognitive change during normal aging, it is well-known that estimates of performance on cognitive tasks are different when calculated using cross-sectional vs. longitudinal data (e.g., Unger et al., 1999; Sliwinski and Buschke, 1999; Salthouse et al., 2004). Additionally, it has been reported that task experience, as well as maturation, may affect test results at 1-year retest intervals (Anderson et al., 2001). Given that neuropsychological models often characterize cognitive development in terms of age in years, it would be useful to compare annual estimates of cognitive development derived from longitudinal and cross-sectional study designs to identify whether these methods give similar results or if there are discernable differences in these approaches.

In a series of small cross-sectional studies we found that the tasks from the Cogstate Brief Battery (CBB), developed primarily for adults, were acceptable unchanged in children as young as 10 years of age (Mollica et al., 2004; Cairney et al., 2007; Collie et al., 2007; Maruff et al., 2009; Lewis et al., 2010). The CBB includes tasks of simple reaction time (Detection task), choice reaction time (Identification task), one-back working memory (One Back task), and continuous recognition visual learning (One Card Learning task). Simple reaction time tasks have a long history in both experimental psychology and neuropsychology and measure how quickly an individual can respond to a stimulus (Verhaeghen and Cerella, 2002; Salthouse and Davis, 2006). Thus, simple reaction time tests are used as a measure of psychomotor function and processing speed. In contrast, performance on choice reaction time tasks requires greater processing time due to their increased demands on attention and perceptual abilities. Therefore, choice reaction time tests can be used to measure overt attention (Luce, 1986). N-back tasks require individuals to maintain information in working memory for a brief time (Shallice et al., 2002). The simplest condition of the n-back paradigm (i.e., one-back) is most commonly used to model working memory in functional imaging (fMRI) and electroencephalogram (EEG) studies (Cohen and Leckman, 1994; Owen et al., 1997; Jansma et al., 2000; Deiber et al., 2007). Continuous visual recognition learning paradigms are also used frequently in fMRI studies of memory or in cognitive psychological studies of aging (Salthouse and Davis, 2006; Squire and Kandel, 2008). These tasks requires individuals to learn a set of stimuli on the basis of their serial and repeated exposure. Learning is operationally defined as the ability to discriminate between learned (i.e., stimuli seen previously) and novel (i.e., distractors) information and involves pattern separation (Yassa and Stark, 2011). Thus, the tasks used in the CBB can be considered to be measures of psychomotor function, attention, working memory, and visual learning. Furthermore, the design of the CBB, in which the stimuli and response requirements for each task are the same, mean that the different cognitive constructs are measured with equivalent perceptual, motor, and linguistic demands. These conditions render the CBB tasks as acceptable, reliable and valid for comparing multiple cognitive constructs across children of different ages as well in adults.

The aim of the current study was to characterize maturational changes in cognition across adolescence using the CBB. We hypothesized that cognitive development from late childhood into early adulthood would follow a negatively decelerating trend in which rates of improvement in performance decreased with increasing age (Luna et al., 2004; Luna, 2009). We expected that lower order cognitive domains (i.e., psychomotor function and attention) would mature earlier than higher order domains (i.e., working memory and learning), but that the rank order for required processing time for each of these cognitive functions would remain the same throughout development (i.e., from fastest to slowest reaction time, the order of cognitive tasks would always be: psychomotor function, attention, working memory, and visual learning). Based on previous results with the CBB, we also anticipated that any observed changes in these cognitive domains over time would not be modulated by gender (Lewis et al., 2010). Finally, we compared results from cross-sectional and longitudinal data to determine if these approaches aligned or if they produced different results.

## Material and methods

### Samples

Two samples were examined in this study, a cross-sectional sample and a longitudinal sample. Both samples consisted of adolescents between the ages of 10 and 18 years old who completed cognitive testing on the Cogstate Brief Battery (CBB) as part of their involvement in concussion management programs in which the CBB was used to conduct baseline cognitive assessments. All individuals, or where appropriate their parent or guardian, agreed to the terms of a privacy policy that allowed the aggregation of their de-identified data for research purposes.

#### Cross-sectional sample

The cross-sectional sample consisted of 38,778 American individuals aged 10–18 years who were separated into single year age cohorts. A single baseline cognitive test from each individual was examined. This was always the first baseline test taken by the individual that met CBB integrity criteria (i.e., was deemed an acceptable test).

#### Longitudinal sample

The longitudinal sample consisted of 5788 American individuals aged 10–18 years. Each of these individuals completed two acceptable CBB assessments approximately 1 year apart. This sample was also separated into single year age cohorts; for the purposes of being placed into a cohort, each individual's age was determined at the time of their first test. The second test was completed from 10 to 14 months after the first test (i.e., the “1-year repeat assessment”).

### Procedure

#### Tasks

The CBB consists of four tasks: Detection (DET; Psychomotor Function), Identification (IDN; Attention), One Back (OBK; Working Memory), and One Card Learning (OCL; Visual Learning). At the start of the CBB assessment, individuals learn to respond using the “Yes” and “No” response buttons. In this sample, these corresponded to the “K” and “D” keys on the keyboard, with “K” used to respond “Yes” and “D” used to respond “No.” All four CBB tasks follow the same general format. Task instructions are provided first and individuals then start by viewing the top of a deck of playing cards on their computer screen. The cards all start face down. As soon as the top card of the deck flips over revealing its face (e.g., Ace of diamonds, 10 of hearts, 8 of spades, etc.), the individual must respond “Yes” or “No” as quickly and accurately as possible depending on the particular task's instructions. After an individual responds, the face up card then flips away from the deck revealing the back of the next card (i.e., the next trial). If the individual answered correctly, the card flips off the top of the deck to the right. Conversely, for incorrect responses, the card flips to the left. Audio feedback also indicates if a response was correct or incorrect. All four CBB tasks record both speed and accuracy data on every trial.

#### Detection task (DET; psychomotor function)

The DET task is a measure of psychomotor function (information processing speed) and uses a well-validated simple reaction time paradigm. Psychomotor function is defined as the average speed by which an individual can initiate a motor action (i.e., key press) in response to a visual stimulus. A single playing card (i.e., the joker) is used as the stimulus in this task. The instructed question is “Has the card turned over?” Individuals must respond with a press of the “Yes” button as soon as they detect the card flip, but not before. A “Yes” response is the only possible output.

#### Identification task (IDN; attention)

The IDN task measures attention and uses a well-validated choice reaction time paradigm. Individuals must attend to stimulus color and then make a differential motor response (i.e., pick the appropriate button) depending on the color of the stimulus. The IDN task is similar to DET, however, there are two possible stimuli which are either a red or black joker card. The individual is asked whether the card currently being presented in the center of the screen is red. The individual should respond by pressing the “Yes” key when the joker card is red and the “No” key when it is black.

#### One back task (OBK; working memory)

The OBK task is a measure of working memory and uses a well-validated n-back paradigm. Individuals are required to hold in mind the image of the last item they saw and compare the memory of this image to the next stimulus. This task uses any of the 52 standard playing cards in a deck as possible stimuli, but no jokers. The individual is asked, “Is this card the same as the previous card?,” and must respond “Yes” when the card they are viewing matches the previous card (e.g., an Ace of Hearts is shown and the last card shown was also an Ace of Hearts) but “No” if the card does not match the previous card (e.g., the individual is viewing an Ace of Hearts but the card before was an Ace of Diamonds).

#### One card learning task (OCL; visual learning)

The OCL task measures visual learning and uses a well-validated pattern separation paradigm. This task also uses the full 52 standard playing cards as possible stimuli. Individuals are asked “Have you seen this card before?” in the current test. They must respond “Yes” if the card they are currently viewing has been shown previously during the test or “No” if the card has not been shown before. For instance, if the first three cards shown in order were: (1) Ace of hearts, (2) King of clubs, (3) Ace of hearts, the corresponding correct responses would be (1) No, (2) No, (3) Yes. The yes response occurred when the Ace of hearts (in position #3) was seen before (in position #1).

#### Outcome measures

The CBB captures both speed and accuracy outcome measures for each of the tasks. Speed of responses is calculated by computing the mean of the individual log10 transformed reaction times of each correct response for each task. Correct responses following anticipations (responses prior to the card face being visible) are excluded from this calculation since these are known to be slower compared to typical (i.e., not post-anticipatory) correct responses. Accuracy is computed by taking the arcsine square root of the proportion of correct responses for each task.

### Data analysis

Data were analyzed using the Statistical Package for the Social Sciences (SPSS) for Windows version 18 and Matlab version R2013b with the Curve Fitting and Statistical toolboxes. Preliminary analysis was conducted to test the assumptions underlying the parametric statistical procedures used. The analyses then proceeded in five steps.

First, data from each individual were grouped into 1-year cohorts based on the age of the individual at the time of testing. Second, descriptive statistics were computed for speed and accuracy for each task within the CBB. Third, the relationship between age and performance was modeled for the cross-sectional sample by fitting either exponential or linear curves to the mean of each age cohort for both (1) age by speed and (2) age by accuracy relationships. These fits were generated using the Matlab curve fitting tool and chosen based on goodness-of-fit statistics (*r*^2^). The parameters of these fits and their 95% confidence intervals were used to describe relationships across tasks throughout maturation. The fit analyses were done with the entire cross-sectional sample combined and also comparing males to females from the cross-sectional sample to examine the effect of gender. Fourth, to further characterize the extent to which performance on these tasks changed from year to year, effect sizes were computed for the difference between means for adjacent age groups (Cohen, 1988). Fifth, we repeated this analysis with the longitudinal sample focusing on actual annual changes in the same individuals based on the 1-year repeat assessment. Since the longitudinal sample contained repeated measures, we calculated these effect sizes based on Dunlap's d (Dunlap et al., 1996).

## Results

### Developmental trajectories on the cogstate brief battery

**Figure 1:**
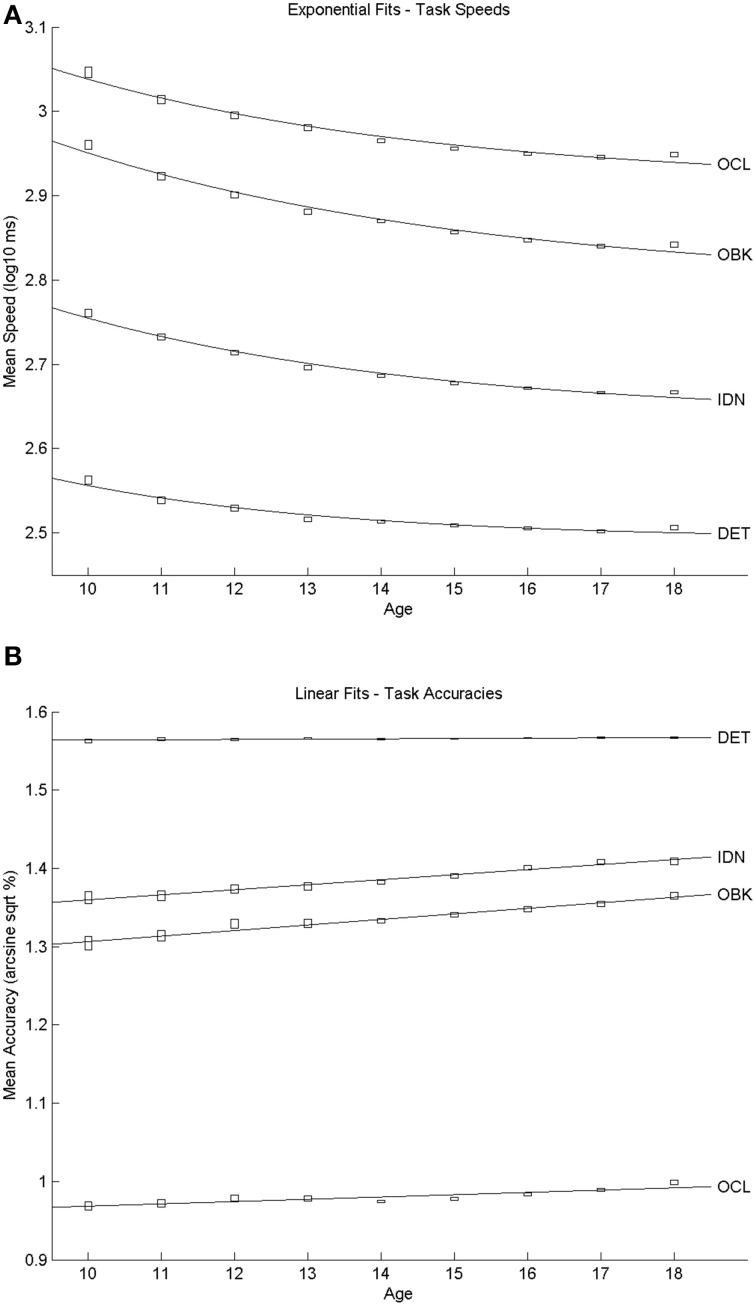
**Developmental trajectories for speed (A) and accuracy (B) by age relationships on each of the CBB tasks**. Speed results were best fit by exponential equations whereas accuracy results were best fit by linear equations. The center of the “rectangle” representing each datapoint indicates the mean and the height of the rectangle indicates the 95% confidence intervals around the mean.

The size of the cross-sectional sample is summarized in Table 1 and shows that this sample exceeded 1000 individuals per cohort. The sample was comprised of 67% males and 33% females. Scatter plots of the relationship between mean speed of performance and age are presented in Figure 1A. The curve fitting analyses indicated that the relationship between performance speed and age was best explained by a negative exponential curve for each task. All exponential fits yielded very high goodness-of-fit statistics (*r*^2^ ≥ 0.96; see Table 2). Similarly, scatter plots of the relationship between mean performance accuracy and age are shown in Figure 1B. These trajectories were well-represented by linear fits. The fit statistics were highest for the IDN and OBK tasks (*r*^2^ ≥ 0.97; see Table 2), though the DET and OCL task fits (*r*^2^ = 0.75 and 0.76, respectively) were still quite good. Figure 1B suggests that the lower estimate of variation explained by the linear relationship between age and performance accuracy for the DET task may be due to performance levels occurring near the maximum possible score on the task across all ages (max = 100%, arcsine sqrt transformed = 1.5708). Thus, the slope of the linear relationship for DET was essentially flat (*m* = 0.0004, see Table 2).

**Table 1:** **Sample sizes for each age group in the cross-sectional and longitudinal samples**.

**Age**	**Cross-sectional**			**Longitudinal**
	**Sample size (*n*)**	**Males (*n*)**	**Females (*n*)**	**Sample size (*n*)**
10	1085	929	156	102
11	1548	1174	374	216
12	2134	1495	639	285
13	2785	1874	911	227
14	7876	5150	2726	1521
15	7708	4960	2748	1574
16	6541	4386	2155	1272
17	5986	4094	1892	274
18	3115	2039	1076	317
Total	38,778	26,101	12,677	5788

*For the longitudinal sample, the age listed is the age at the first test. A second test was taken 1 year later*.

**Table 2:** **Best fit equations for speed (exponential) and accuracy (linear) and goodness-of-fit statistics (*r*^2^)**.

**Task**	**Equation**	***r*^2^**
Speed	*y* = exp(m^*^x) + b	
DET	*y* = exp(−0.26^*^x) + 2.49	0.96
IDN	*y* = exp(−0.20^*^x) + 2.64	0.99
OBK	*y* = exp(−0.18^*^x) + 2.80	0.98
OCL	*y* = exp(−0.20^*^x) + 2.91	0.98
Accuracy	*y* = m^*^x + b	
DET	*y* = 0.0004^*^x + 1.56	0.75
IDN	*y* = 0.0064^*^x + 1.29	0.97
OBK	*y* = 0.0071^*^x + 1.23	0.98
OCL	*y* = 0.0029^*^x + 0.94	0.76

The characteristics of the curves were then compared to determine the extent to which performance was influenced by task complexity or age-related factors. Each fit model contained two parameters, a rate of change term (“*m*”) and an offset term (“*b*”). These are slope and intercept terms, respectively for the linear fit and describe similar characteristics for the exponential fits. The fit equations with resulting coefficient terms for each task for speed and accuracy are shown in Table 2.

**Figure 2:**
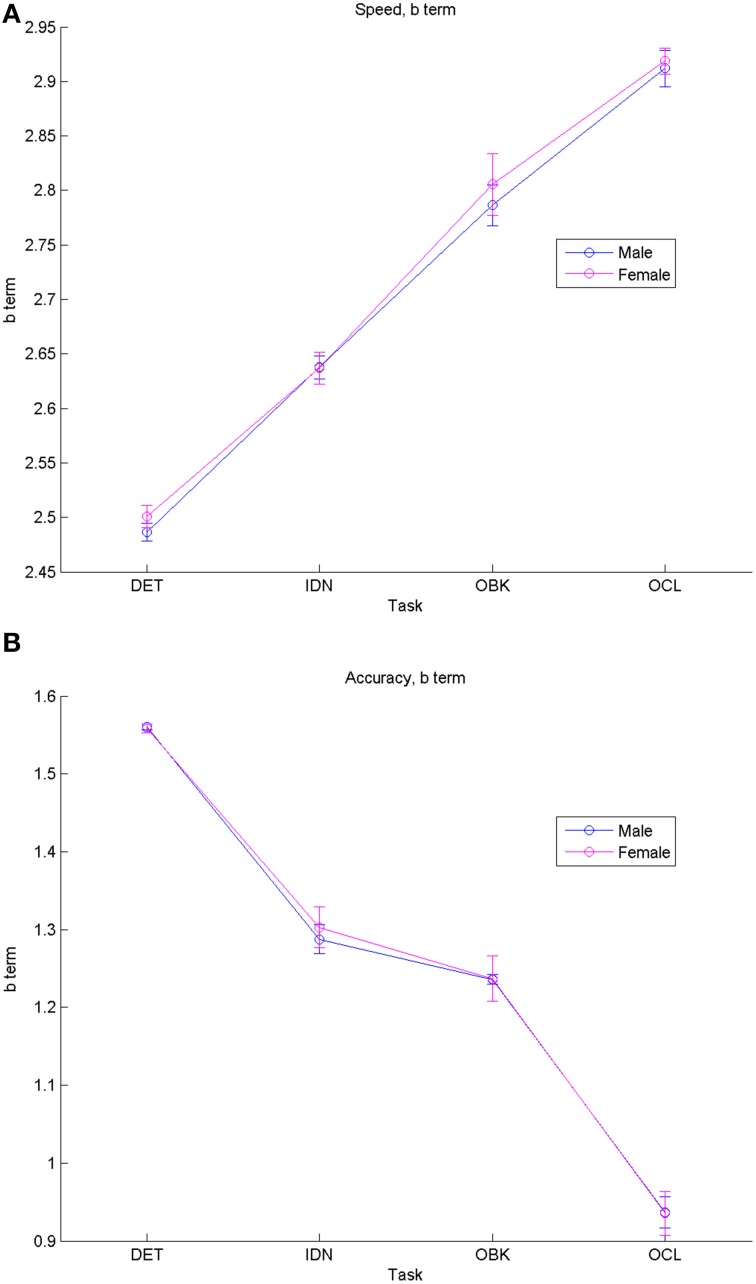
**Comparisons of fit equation “*b*” (“offset”) terms for males vs. females show similar increases in speed (i.e., longer reaction times) (A) and decreases in accuracy (B) as task difficulty increases for both genders**. Error bars represent 95% confidence intervals for the fitted model.

Figure 2 plots fit model “*b*” terms and their 95% CIs for each task when segregated by gender for both speed (Figure 2A) and accuracy (Figure 2B). These data indicate that performance was similar between males and females (i.e., there was overlap between the 95% CIs for the model fits). However, there were significant offsets for both speed and accuracy across each task based upon the task complexity. The tasks from easiest to hardest are DET (psychomotor function), IDN (attention), OBK (working memory), and OCL (visual learning). Speed *b* coefficients increased linearly with task difficulty, with larger offset (*b*) terms for harder tasks corresponding to the slower reaction times occurring during these tasks. Likewise, accuracy performance declined as task difficulty increased with the highest score seen on DET (i.e., the easiest task) and the lowest score seen on OCL (i.e., the hardest task).

**Figure 3:**
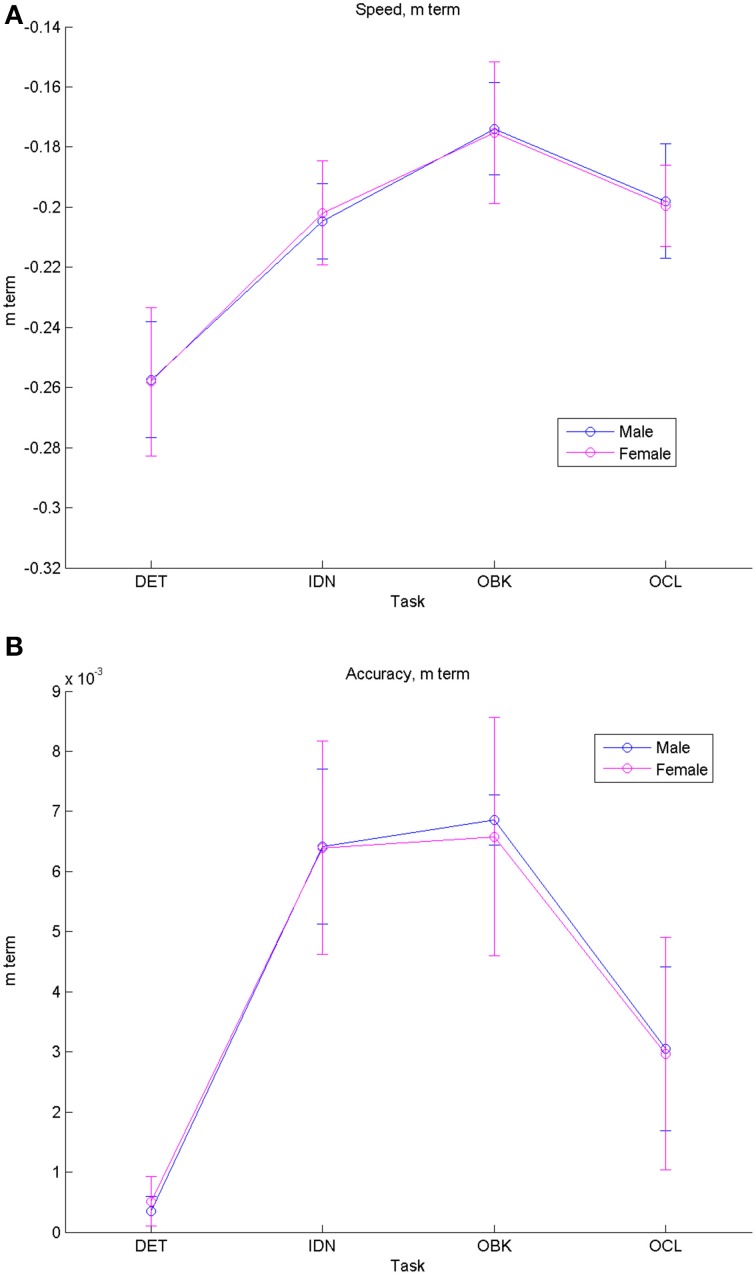
**Comparisons of fit equation “*m”* (“rate of change”) terms for males vs. females show similar developmental rates of change for both genders in speed (A) and accuracy (B)**. Error bars represent 95% confidence intervals for the fitted models.

Figure 3 shows the group model “*m*” terms and their 95% CIs which describe the rate of change across development for each task separated by gender. These data also indicate similar performance by males and females on the CBB tasks. Speed improved with maturation at a similar rate of change for the IDN, OBK, and OCL tasks, but improved at a slower rate for the DET (psychomotor function) task (Figure 3A). Accuracy showed little improvement with maturation for the DET task since all age groups were near maximal performance on that task (Figure 3B). Accuracy improved with maturation at similar rates for IDN (attention) and OBK (working memory), but improved more slowly over time for the OCL (visual learning) task.

The results of separate curve fits based on gender suggest there is no gender effect on the CBB tasks as model fits for both males and females always had overlapping fit confidence intervals. We tested this further statistically by drawing random samples of matched size (*n* = 125) for both males and females from each age group. These results are shown for speed (**Table 4**) and accuracy (**Table 5**). Omnibus significance tests for each age cohort across each task for speed and accuracy confirmed no significant gender effect even at nominal levels (*t*-test, *p* > 0.01). Effect size differences for males and females were also uniformly low (mean *d* = 0.07, std *d* = 0.12, max *d* = 0.27) and effect size 95% confidence intervals almost always overlapped with zero (**Tables 4**, **5**). Based on these results indicating similar performance across gender, we examined age-related improvement on the CBB from the full dataset without consideration for gender going forward.

### Annual change in performance

**Figure 4:**
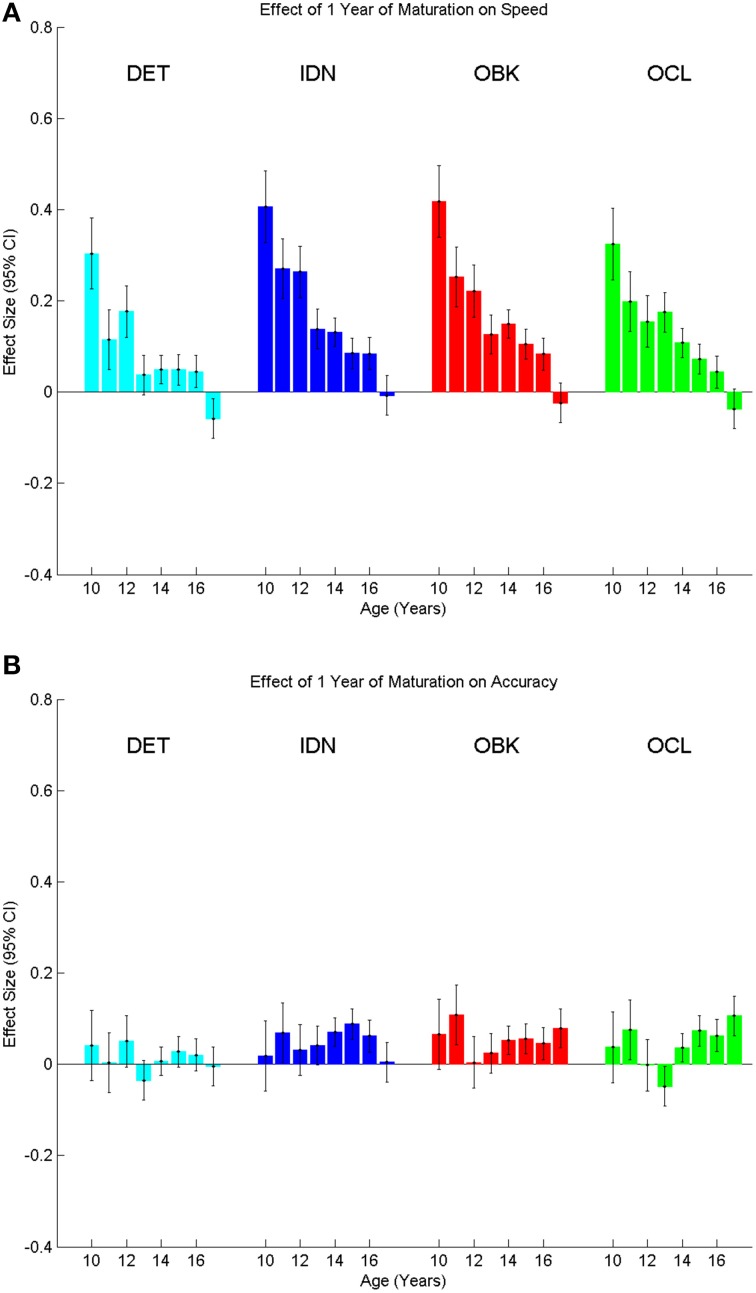
**Effect sizes for speed (A) and accuracy (B) comparing the change between adjacent age years for each task in the CBB**. The bar at each age represents the effect size of the difference in performance between that age and the age 1 year older (e.g., bars at age 10 represent ages 10 vs. 11). Error bars indicate 95% confidence intervals for the effect size.

The means for performance speed and accuracy of age cohort, along with the standard deviations in performance, are provided in Table 3. Inspection of these data show that while both mean speed and accuracy change with age, estimates of variation for each task remained approximately equivalent across ages. We therefore computed Cohen's d effect sizes to examine the magnitude of change in performance on the CBB tasks with age. We first examined annual change in performance for spee d, by computing effect sizes for adjacent year pairs (e.g., 10 vs. 11, 11 vs. 12, etc.). Improvement occurred at the greatest magnitude for the younger ages (Figure 4A). This corresponds to the steep slope of the exponential fits shown in Figure 1. As age increased, the magnitude of change seen in a single year diminished, with the 95% confidence intervals of the effect size generally overlapping with zero between the ages of 17 and 18 years. Figure 4B similarly shows annual effect sizes for accuracy. Effect size changes in accuracy per annum on each task were uniformly very small (i.e., *d* < 0.1)

**Table 3:** **Group means and standard deviations for the speed and accuracy of performance on each task in the CBB for each year of age between 10 and 18 years**.

**Age**	***N***	**DET**		**IDN**		**OBK**		**OCL**	
		***M***	***SD***	***M***	***SD***	***M***	***SD***	***M***	***SD***
**SPEED**									
10 years	1085	2.56	0.08	2.76	0.07	2.96	0.09	3.05	0.11
11 years	1548	2.54	0.08	2.73	0.07	2.92	0.09	3.01	0.10
12 years	2134	2.53	0.08	2.71	0.07	2.90	0.09	3.00	0.09
13 years	2785	2.52	0.08	2.70	0.07	2.88	0.09	2.98	0.09
14 years	7876	2.51	0.08	2.69	0.07	2.87	0.09	2.97	0.09
15 years	7708	2.51	0.08	2.68	0.07	2.86	0.09	2.96	0.08
16 years	6541	2.51	0.08	2.67	0.07	2.85	0.09	2.95	0.09
17 years	5986	2.50	0.08	2.67	0.06	2.84	0.09	2.95	0.08
18 years	3115	2.51	0.07	2.67	0.06	2.84	0.09	2.95	0.08
Total	38,778								
**ACCURACY**									
10 years	1085	1.56	0.04	1.36	0.13	1.30	0.15	0.97	0.09
11 years	1548	1.56	0.04	1.36	0.12	1.31	0.14	0.97	0.09
12 years	2134	1.56	0.03	1.37	0.12	1.33	0.14	0.98	0.09
13 years	2785	1.57	0.03	1.38	0.12	1.33	0.13	0.98	0.09
14 years	7876	1.57	0.03	1.38	0.12	1.33	0.14	0.97	0.09
15 years	7708	1.57	0.03	1.39	0.12	1.34	0.14	0.98	0.09
16 years	6541	1.57	0.03	1.40	0.12	1.35	0.14	0.98	0.09
17 years	5986	1.57	0.03	1.41	0.12	1.35	0.13	0.99	0.09
18 years	3115	1.57	0.03	1.41	0.12	1.37	0.13	1.00	0.09
Total	38,778								

**Figure 5:**
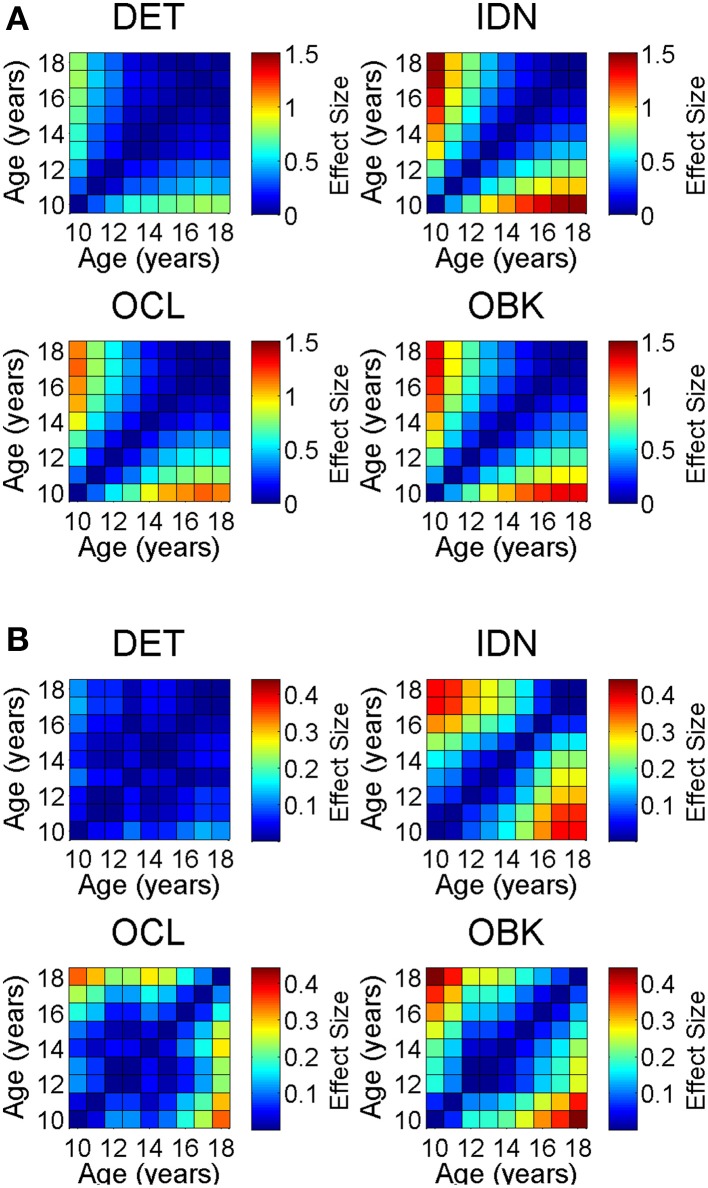
**Correlation matrices showing the effect size on speed (A) and accuracy (B) of change in cognition across any combination of ages between 10 and 18 for each task in the CBB**. The diagonals represent the unity line where the same age is compared with itself and therefore all effect sizes on the diagonal are zero (e.g., age 10 vs. 10). Color scales representing effect size magnitude are different on **(A)** vs. **(B)** indicating larger effect sizes for speed vs. accuracy.

We next plotted correlation matrices that allowed comparison of the effect size across any combination of age cohorts in the sample for both speed and accuracy for each task (Figure 5). The magnitude of the effect size is represented by the color of each location in the matrices. The center diagonal of each matrix is the unity line (e.g., comparing the same age against itself) and therefore the effect size is always 0. The full correlation matrices show that while the effect size of a single year of maturation was typically relatively small on the CBB tasks (as seen also in Figure 4), the effect greatly increases as the separation between age cohorts being compared increases (i.e., as you move away from the unity line). Effect sizes for speed (Figure 5A, max *d* = 1.46) were much larger than those for accuracy (Figure 5B, max *d* = 0.44).

**Figure 6:**
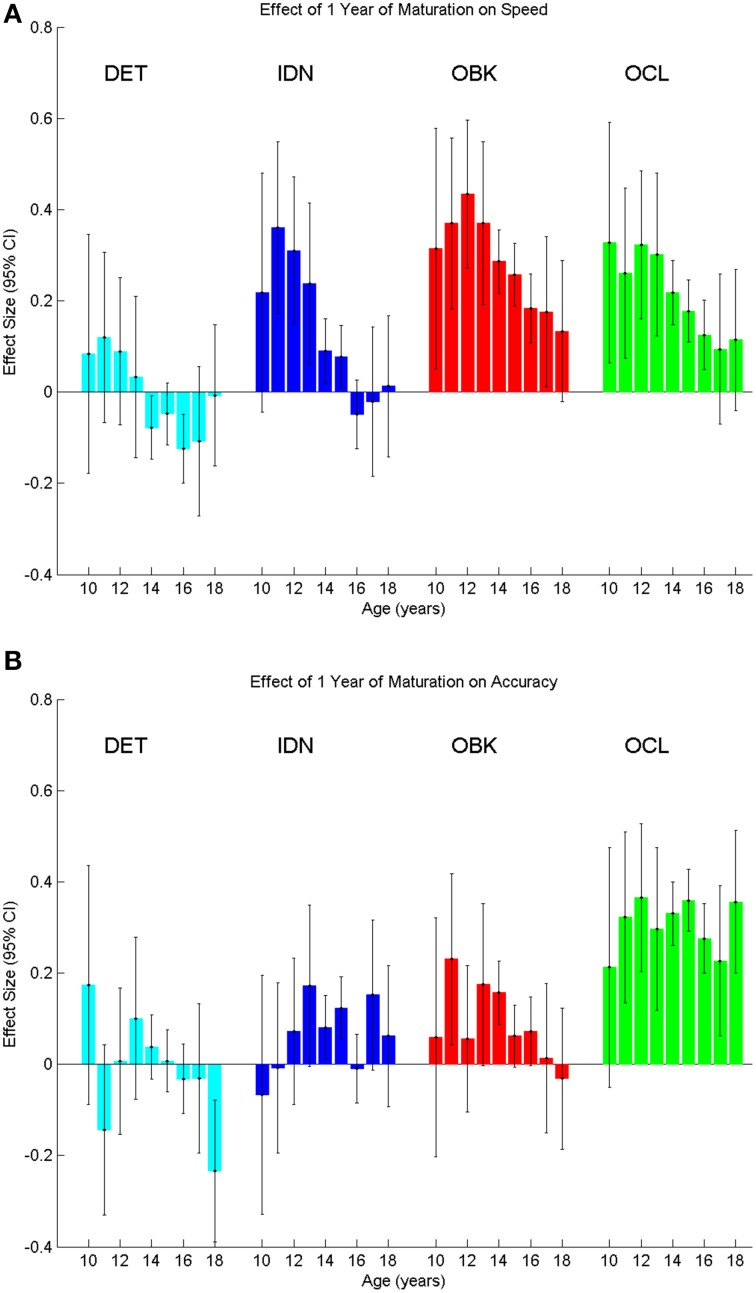
**Yearly effect size plots for speed (A) and accuracy (B) compare the change between a first and second test on the CBB taken 1 year apart in the same individuals (longitudinal data)**. Error bars represent 95% confidence intervals for the effect size.

To verify that the cross-sectional data were reflective of true change in cognition within a given individual, we next analyzed the longitudinal sample, which contained two tests per individual with the second test administered 1 year after the first. The sample size for each age cohort from 10 to 18 years old is reported in Table 1. One-year effect size comparisons of the longitudinal data (Figure 6) were generally consistent with those from the cross-sectional data (Figure 4). Speed measures still had the largest effect sizes for younger age groups and overall the magnitude of the effect size again decreased with age across all tasks (Figure 6A). Likewise, accuracy effect sizes were typically small across all ages (Figure 6B).

Accuracy for the OCL task was the only instance in which the longitudinal data differed from the cross-sectional data. In this case, medium effect sizes approaching 0.4 occurred for the majority of ages. Further analysis into this difference showed that the second longitudinal assessments (i.e., the 1-year repeat assessments) for OCL were always significantly better than the first assessments for the OCL of the next age cohort (*t*-test, *p* < 0.05). For example, repeat assessments of the 10 year old age cohort, taken when these individuals were 11 years old, were significantly better than the initial assessments of the 11 year old age cohort, taken when these individuals were 11 years old.

## Discussion

This study characterized the nature and rate of cognitive change across adolescent development. Examination of performance on the CBB in a large, cross-sectional sample of individuals aged 10–18 years revealed that speed of performance on each CBB task improved with age and this was fit most accurately as a negative exponential decrease in reaction time with increasing age. The nature of the age-related improvement in speed of performance was qualitatively similar for each CBB task (Figure 1A) and in all cases fit exceptionally well by the same exponential model (e.g., *r*^2^'s > 0.96; Table 2). Differences between tasks in the exponent term used to characterize the age-related improvement in performance speed showed that the improvement was slightly less for speed of psychomotor function (DET task) than it was for the three other cognitive functions measured by the CBB tasks. No differences in age-related improvement in performance speed were observed for the remaining three tasks based on overlapping 95% confidence intervals of the model fits (Figure 3A). As expected, speed of performance was slower with increasing difficulty of the CBB tasks. This was illustrated both in the offset of the curves across tasks (Figure 1A) as well as in the differences between tasks in the offset terms derived from the relationship between performance speed and age (Figure 2A).

As hypothesized, the nature of speed-related change across adolescent development was not linear, but instead slowed with increasing age. Correspondingly, effect sizes for 1-year age differences (Figure 4A) were largest at younger ages when cognition was changing more rapidly and diminished until effect size confidence intervals overlapped with zero between ages 17 and 18 years, suggesting that, at this point, the cognitive constructs measured by these tests had stopped improving. Effect sizes were smaller for psychomotor function (DET task) relative to the other cognitive constructs measured and stopped improving at a younger age, first overlapping with zero between ages 13 and 14 years. This result, combined with the slower rate of improvement seen across adolescent maturation on the DET task, suggests that psychomotor functioning (i.e., simple reaction time) may mature sooner than attention, working memory, and learning. Finally, speed model fits were similar between males and females (Figures 2A, 3A) and no significant differences were seen in performance speed based on gender during maturation (Table 4).

**Table 4:** **Speed data by task from random samples of 125 males and females of each age group**.

**Task**	**Age**	**Female Mean**	**Male Mean**	**Female SD**	**Male SD**	***p***	***d***	**d LL**	**d UL**
DET	10	2.57	2.56	0.07	0.07	0.23	0.14	−0.10	0.39
DET	11	2.56	2.54	0.08	0.08	0.06	0.23	−0.02	0.48
DET	12	2.55	2.53	0.08	0.08	0.05	0.23	−0.02	0.47
DET	13	2.53	2.51	0.08	0.08	0.03	0.27	0.02	0.52
DET	14	2.52	2.51	0.07	0.08	0.18	0.19	−0.06	0.43
DET	15	2.52	2.51	0.09	0.08	0.27	0.14	−0.10	0.39
DET	16	2.52	2.50	0.08	0.07	0.09	0.23	−0.02	0.48
DET	17	2.51	2.51	0.07	0.08	0.46	0.09	−0.15	0.34
DET	18	2.52	2.51	0.07	0.07	0.50	0.09	−0.16	0.34
IDN	10	2.77	2.76	0.06	0.07	0.76	0.04	−0.21	0.29
IDN	11	2.74	2.74	0.06	0.08	0.54	−0.07	−0.32	0.18
IDN	12	2.71	2.72	0.06	0.07	0.91	−0.01	−0.26	0.23
IDN	13	2.70	2.69	0.08	0.07	0.78	0.03	−0.21	0.28
IDN	14	2.70	2.69	0.07	0.07	0.21	0.17	−0.08	0.41
IDN	15	2.67	2.67	0.08	0.07	0.81	0.03	−0.22	0.28
IDN	16	2.68	2.67	0.07	0.07	0.36	0.12	−0.13	0.37
IDN	17	2.67	2.68	0.06	0.07	0.31	−0.13	−0.37	0.12
IDN	18	2.66	2.68	0.06	0.07	0.08	−0.22	−0.47	0.03
OBK	10	2.99	2.97	0.09	0.10	0.06	0.24	−0.01	0.49
OBK	11	2.93	2.92	0.09	0.08	0.11	0.20	−0.05	0.45
OBK	12	2.92	2.90	0.08	0.09	0.15	0.18	−0.06	0.43
OBK	13	2.89	2.87	0.10	0.09	0.15	0.18	−0.07	0.43
OBK	14	2.88	2.88	0.08	0.10	0.74	0.05	−0.20	0.29
OBK	15	2.86	2.85	0.10	0.09	0.72	0.04	−0.20	0.29
OBK	16	2.86	2.84	0.09	0.09	0.10	0.21	−0.04	0.46
OBK	17	2.86	2.84	0.08	0.10	0.07	0.23	−0.02	0.48
OBK	18	2.85	2.84	0.09	0.10	0.23	0.14	−0.11	0.39
OCL	10	3.06	3.04	0.10	0.10	0.34	0.12	−0.12	0.37
OCL	11	3.01	3.02	0.10	0.09	0.48	−0.09	−0.34	0.16
OCL	12	3.01	2.99	0.08	0.10	0.37	0.11	−0.14	0.36
OCL	13	2.98	2.98	0.09	0.09	0.62	0.06	−0.19	0.31
OCL	14	2.98	2.97	0.09	0.10	0.46	0.09	−0.15	0.34
OCL	15	2.96	2.95	0.09	0.08	0.12	0.20	−0.05	0.45
OCL	16	2.96	2.95	0.08	0.09	0.36	0.12	−0.13	0.37
OCL	17	2.96	2.97	0.08	0.11	0.53	−0.08	−0.33	0.17
OCL	18	2.96	2.96	0.09	0.08	0.88	−0.02	−0.27	0.23

A similar pattern was observed for performance accuracy. With the exception of the DET (psychomotor function) task, where accuracy of performance was close to error-free for all ages studied, mean performance accuracy improved with increasing age. The nature of the age-related improvement was qualitatively similar for each of the other tasks (Figure 1B). For each CBB task, age-related improvement in performance accuracy was best described by a linear function. Comparisons of the slope of the linear fits describing age-related improvement in performance accuracy indicated a slower rate of improvement for the OCL (visual learning) task and faster rates of improvement for the IDN (attention) and OBK (working memory) tasks (Figure 3B). Once again, task difficulty influenced the overall accuracy on each CBB task with the offsets of the linear functions between age and performance accuracy decreasing with task difficulty (Figure 2B). Likewise, no differences between adolescent males and females were evident in the developmental trajectory fits (Figures 2B, 3B) nor were there any statistically significant effects of gender across task or age (Table 5). Changes in accuracy on the CBB across adolescent development were much smaller than those seen for speed (Figures 4, 5) and were of small effect size on a single year basis (Figure 4B). However, when examined across larger age separations (e.g., several years apart) the effect on accuracy was more pronounced (Figure 5B).

**Table 5:** **Accuracy data by task from random samples of 125 males and females of each age group**.

**Task**	**Age**	**Female Mean**	**Male Mean**	**Female SD**	**Male SD**	***p***	***d***	**d LL**	**d UL**
DET	10	1.56	1.56	0.05	0.06	0.44	0.09	−0.16	0.34
DET	11	1.56	1.57	0.05	0.04	0.41	−0.11	−0.35	0.14
DET	12	1.56	1.56	0.05	0.04	0.30	−0.13	−0.38	0.11
DET	13	1.57	1.56	0.03	0.04	0.51	0.09	−0.16	0.33
DET	14	1.57	1.56	0.02	0.05	0.10	0.21	−0.04	0.46
DET	15	1.57	1.56	0.03	0.05	0.33	0.11	−0.14	0.36
DET	16	1.57	1.57	0.03	0.02	0.43	−0.10	−0.35	0.15
DET	17	1.57	1.57	0.02	0.02	0.99	0.00	−0.25	0.25
DET	18	1.57	1.57	0.01	0.03	0.27	0.14	−0.11	0.39
IDN	10	1.39	1.37	0.13	0.13	0.11	0.20	−0.05	0.45
IDN	11	1.37	1.35	0.13	0.12	0.09	0.22	−0.03	0.47
IDN	12	1.38	1.38	0.12	0.11	0.92	0.01	−0.23	0.26
IDN	13	1.40	1.38	0.12	0.12	0.10	0.21	−0.04	0.46
IDN	14	1.38	1.37	0.12	0.13	0.50	0.09	−0.16	0.33
IDN	15	1.40	1.39	0.13	0.11	0.55	0.08	−0.17	0.33
IDN	16	1.41	1.41	0.12	0.13	0.76	0.04	−0.21	0.29
IDN	17	1.43	1.40	0.13	0.13	0.05	0.24	0.00	0.49
IDN	18	1.43	1.42	0.12	0.12	0.52	0.08	−0.17	0.33
OBK	10	1.28	1.31	0.13	0.15	0.08	−0.23	−0.48	0.02
OBK	11	1.29	1.32	0.14	0.15	0.14	−0.17	−0.42	0.08
OBK	12	1.34	1.33	0.14	0.14	0.58	0.07	−0.18	0.32
OBK	13	1.34	1.34	0.13	0.15	0.92	−0.01	−0.26	0.24
OBK	14	1.33	1.33	0.13	0.14	0.88	−0.02	−0.27	0.23
OBK	15	1.35	1.34	0.13	0.13	0.49	0.08	−0.16	0.33
OBK	16	1.36	1.35	0.13	0.14	0.58	0.07	−0.18	0.32
OBK	17	1.34	1.34	0.14	0.13	0.85	0.02	−0.23	0.27
OBK	18	1.35	1.36	0.12	0.14	0.54	−0.08	−0.33	0.17
OCL	10	0.96	0.98	0.09	0.10	0.12	−0.19	−0.44	0.06
OCL	11	0.98	0.97	0.09	0.08	0.91	0.02	−0.23	0.26
OCL	12	0.97	0.98	0.08	0.09	0.46	−0.09	−0.34	0.16
OCL	13	0.99	0.98	0.09	0.08	0.29	0.14	−0.11	0.39
OCL	14	0.96	0.97	0.09	0.08	0.71	−0.05	−0.29	0.20
OCL	15	0.98	0.98	0.08	0.09	0.69	0.05	−0.20	0.30
OCL	16	0.99	0.99	0.09	0.08	0.49	0.09	−0.16	0.34
OCL	17	0.99	0.99	0.09	0.10	0.74	0.04	−0.21	0.29
OCL	18	1.01	1.01	0.09	0.09	0.94	−0.01	−0.26	0.24

The results of this study characterize the rates of improvement of various cognitive domains across adolescent maturation and establish age appropriate norms for the tasks of the CBB. Reaction time improvements were of larger magnitude than accuracy improvements, but the rate of improvement for speed slowed with aging whereas this was not the case for accuracy. As hypothesized, while the exact rate of improvement did differ somewhat across tasks (i.e., across cognitive domains), the relative order of task difficulty remained constant across maturation. That is, reaction times were always fastest on DET, followed by IDN, OBK, and finally OCL. Likewise, this rank ordering based on task difficulty was maintained for accuracy as well.

One important implication of these results involves the interpretation of repeated cognitive assessment in adolescence. Since cognitive functioning improves during development, assessments made as an individual matures should take into account the expected rate of improvement found in the cross-sectional data fits. To confirm if this was indeed the case at an individual level, we analyzed a second, longitudinal dataset containing 1-year repeat assessments on the same individuals. These data supported the findings of the cross-sectional analysis. Specifically, true 1-year effect sizes showed the same pattern of improvement in the 1-year repeat assessments (Figure 6) as was seen in the cross-sectional data (Figure 4). The only case where the longitudinal data differed from the cross-sectional data was for the OCL (visual learning) task. The longitudinal data showed that there was better performance on the OCL 1-year repeat assessment than was seen during the cross-sectional cohort of the same age. This suggests that for this task there may be an additional effect beyond that of maturation that is seen during the 1 year repeat assessment. For example, Anderson et al. (2001) identified that task experience could have a greater effect on certain tasks than maturation over a 1 year interval. Furthermore, this effect of task experience was seen to be above and beyond the familiarity or practice effect observed during short-term repeat testing (Anderson et al., 2001). As the OCL task has been reported to have high 1-year test-retest reliability in adults (Louey et al., 2014), it is unclear if there is a long-term task experience effect in children or if some other factor may account for the OCL differences in the cross-sectional and longitudinal data.

While the amount of cognitive change over a single year was typically of low effect size for accuracy (Figures 4B, 6B) and was also of low effect size in later adolescence for speed (Figures 4A, 6A), the magnitude of the effect was much larger across multiple years (Figure 5). As an example, the maximal single year effect size for attention speed (IDN) in the cross-sectional sample was 0.4 between the ages of 10 and 11. However, the effect size increased to 1.5 between ages 10 and 18 years. Thus, neuropsychological evaluations should account for this developmental trajectory when comparing test results to both normative data as well as previous test results during repeated assessment of the same individual.

In addition to providing a firm basis for understanding age-related improvement in performance on the tasks from the CBB, the current data can also inform developmental neuropsychological models of psychomotor function, attention, working memory and learning, at least within the visual domain. For example, we saw continuous improvement across adolescence in all tested cognitive domains. These data fit with the idea that the neural networks for these cognitive functions are in place by the end of childhood, but go through a period of refinement during adolescence such that the networks become more salient by adulthood (Bunge and Wright, 2007). Indeed, the decreasing exponential fits in performance that we observed are similar in time frame to curvilinear changes seen in cortical growth (Raznahan et al., 2011). From 10 to 12 years of age we saw the largest effect sizes of annual change. This corresponds to the period when increasing gray matter in the prefrontal cortex (PFC), the area of the brain responsible for many higher order cognitive abilities, is finishing (Giedd et al., 1999). As this gray matter increase subsides in late childhood, further improvements during adolescence are thought to be due to a combination of synaptic pruning/gray matter decrease and increased myelination (Nagy et al., 2004; Casey et al., 2005; Bunge and Wright, 2007; Luna, 2009).

Our data suggest that while speed of psychomotor function improves at a slightly slower rate during adolescence, the rate of improvement on attention, working memory, and visual learning speed were all similar (Figure 3A). These cognitive domains are associated with the fronto-parietal network where improved white matter formation leads to increased gray matter functioning during this period of development (Nagy et al., 2004). Given the similar reliance of these three cognitive domains on PFC communication with distributed neural circuits (Buschman and Miller, 2007; Cromer et al., 2011; Miller, 2013; Miller and Buschman, 2013), it is not necessarily surprising that they develop at similar rates. Likewise, simple reaction time is less reliant on the PFC and this may account for its slower rate of improvement throughout adolescence, as its primary driving neural mechanisms have likely matured sooner. Differences were also seen in rates of improvement for accuracy performance on the hardest CBB task (OCL) as compared to the other cognitive domains tested. The OCL task is modeled after a pattern separation paradigm (Yassa and Stark, 2011) that is known to be dependent on hippocampal functioning. The hippocampal area is believed to show increases in gray matter volume throughout adolescence (Saitoh et al., 2001), which may account for the slower increase in accuracy rate for the OCL task compared to the other cognitive domains that are less hippocampal dependent.

In summary, we have characterized the developmental trajectories of speed and accuracy improvements in the cognitive domains of psychomotor function, attention, working memory, and visual learning across adolescence. These results inform our understanding of the nature and rate of changes across these cognitive domains relative to one another and establish normative ranges for males and females on the CBB throughout this age range. Maturational effects seen across the various cognitive domains should be taken into account when interpreting cognitive test results during this period of development.

### Conflict of interest statement

Drs. Jason A. Cromer, Adrian J. Schembri, Brian T. Harel, and Paul Maruff are employees of Cogstate, Inc., manufacturer of the Cogstate Brief Battery.
